# Oral Papillary Squamous Cell Carcinoma and Oral Squamous Cell Carcinoma: A Histopathological and Immunohistochemical Comparative Study

**DOI:** 10.1007/s12105-024-01635-4

**Published:** 2024-06-17

**Authors:** Esraa Ashraf Mahmoud, Mohsen Kazem Abdellatif, Sarah Ahmed Mohammed Mahmoud

**Affiliations:** 1 Faculty of Dentistry, Cairo University and Teaching Assistant at the Oral Pathology Department, October University for Modern Sciences and Arts, Cairo, Egypt; 2 Oral and Maxillofacial Pathology, Faculty of Dentistry, Cairo University, Cairo, Egypt

**Keywords:** Oral papillary squamous cell carcinoma, Well-differentiated squamous cell carcinoma, Moderately differentiated squamous cell carcinoma, Poorly differentiated squamous cell carcinoma, Transforming growth factor beta, Alpha smooth muscle actin

## Abstract

**Purpose:**

The aim of the study is to investigate the immunohistochemical expression of both Alpha smooth muscle actin and Transforming Growth Factor beta and compare their expression in oral papillary squamous cell carcinoma with their expression in different histological grades of oral squamous cell carcinoma. A correlation between these immuno-histochemical expressions and histological findings will then be performed. The research question is “Do the percentages of α-SMA and TGF-β immune-expression in OPSCC differ from that in the conventional OSCC?”.

**Methods:**

This will be achieved by collecting archival blocks of oral papillary squamous cell carcinoma and different grades of oral squamous cell carcinoma, staining the specimens with Transforming Growth Factor beta and alpha smooth muscle actin, then measuring the mean staining index of expression in each group and the area percent of both markers.

**Results:**

Results revealed that transforming growth factor beta expression in the epithelium was high in all cases of well-differentiated squamous cell carcinoma, most oral papillary squamous cell carcinoma, and poorly differentiated oral squamous cell carcinoma. On the other hand, different grades of oral squamous cell carcinoma showed a high staining index of alpha smooth muscle actin expression in the stroma. While cases of oral papillary squamous cell carcinoma were either moderate or low-staining.

**Conclusions:**

Oral papillary squamous cell carcinoma has a favourable prognosis compared to different histological grades, and the prognosis does not depend only on histological grade but also on other prognostic factors.

## Introduction

Oral squamous cell carcinoma (OSCC) is considered the most common malignant epithelial neoplasm that affects the oral cavity [1]. The most commonly affected sites for OSCC are the tongue, lips, and floor of the mouth [2]. It can arise either in an apparently normal mucosa or be preceded by one of the oral potentially malignant lesions [3]. On the aspect of histopathology, OSCC is classified into well-differentiated squamous cell carcinoma (WDSCC), moderately differentiated squamous cell carcinoma (MDSCC), and poorly differentiated squamous cell carcinoma (PDSCC) according to their resemblance to the mother cell [4].

One of the rare variants of OSCC is oral papillary squamous cell carcinoma (OPSCC), which was reported to have a favourable prognosis [5]. It has a correlation with the Human Papilloma Virus (HPV), and clinically, it appears as an oral lesion with papillary architecture [6]. It differs from conventional OSCC in that OPSCC has limited invasion, a lower rate of metastasis, and a better survival rate [6].

The prognosis of OSCC can be affected by variable histopathological negative prognostic factors, such as the depth of invasion, the invasive front, worst pattern of invasion (WPOI), bone invasion, tumour-stroma ratio (TSR), and perineural and lymphovascular invasion [7].

Tumor microenvironment (TME) is a dynamic environment that is required for tumor development, and influences tumor prognosis and treatment efficacy. One of the most significant cells in TME are cancer associated fibroblasts (CAFs) [8]. Increased levels of CAFs are associated with advanced T-stage, lymph node (LN) metastasis, vascular invasion, higher rates of perineural invasion, poor differentiation, and a high rate of recurrence [9].

Transforming growth factor beta (TGF-β) is involved in many cellular processes, including proliferation, differentiation, extracellular matrix remodeling, apoptosis, fibrosis, and tumour progression [10]. Once it is elevated, the induction of normal fibroblasts to become CAFs occurs. It alters the epigenetic signature of stromal fibroblasts, resulting in alpha smooth muscle actin (α-SMA) gene expression and increased collagen synthesis in CAFs [11].

Alpha smooth muscle actin (α-SMA), which is the most reliable marker for CAFs, is an actin isoform that is found in smooth muscle cells, myofibroblasts, and blood vessels [12]. It correlates with the activation of fibroblasts into myofibroblasts, or CAFs. As a result, increased α-SMA expression and collagen alteration may predict tumour progression [13].

Therefore, the aim of the study was to investigate the immunohistochemical expression of both α-SMA and TGF-β in OPSCC and compare their expression with different histological grades of OSCC. Then a correlation between the expression of these immunohistochemical markers and histopathological negative prognostic factors was performed. It is believed that OPSCC has a better prognosis than conventional OSCC. Therefore, the expression of α-SMA and TGF-β is expected to show less expression in OPSCC than in conventional OSCC.

## Methodology

### Participants and Staining

Archival blocks of OPSCC and different histological grades of OSCC were collected from Oral and Maxillofacial Pathology, Faculty of Dentistry, Cairo University that fit the histopathological criteria for OPSCC and OSCC as stated in the WHO classification 2022 of head and neck tumors. Cases with an underlying systemic condition were excluded. The cases were examined blindly by 2 different oral pathologists. A total of 60 blocks of OSCC were recruited, where 15 out of 60 cases were OPSCC, 15 out of 60 cases were WDSCC, 15 out of 60 cases were MDSCC, and 15 out of 60 were PDSCC. The specimens were stained histologically with hematoxylin and eosin (H&E) [14], and immunohistochemically with α-SMA and TGF-β [15] at the National Cancer Institute, Cairo University.

### Histopathological Examination for Negative Prognostic Factors

The invasive front was examined in serial sections of each collected case for tumour nests that contained less than five cells, indicating tumour budding. The percentage of tumours with budding was calculated for each group. As for the TSR, the area percent of the stroma in the invasive front was measured in each collected case. If it was more than 50% of the field, the case was considered to have a low TSR. The percentage of tumours with a low TSR was calculated for each group. Last, each collected case was examined thoroughly for perineural and/or perivascular invasion. The percentage of tumours with perineural and/or perivascular invasion was calculated for each group. WPOI 1 was given to cases with well-delineated, pushing, and infiltrative borders, while WPOI 2 was given to cases with an infiltrative finger-like pattern [16]. WPOI 3 was given to small groups of cells greater than 15 tumour cells, WPOI 4 to cases with small groups of less than 15 tumour cells or individual cells, and WPOI 5 to cases with satellite tumour nodules that appeared at least 1 mm away from the main tumour [17].

### Image Analysis

Capturing microscopic images was done in Analytica Research Centre, Elharam, Cairo, Egypt, using a SOPTOP EX20 biological microscope (China), an HD camera (model No. XCAM1080PHB), and Imageview software at X40, X100, and X200 magnification powers. Immuno-stained sections were examined using high-power fields (X400), and the most homogenous areas of the positive reaction were chosen for evaluation.

### Staining Index of Immunohistochemical Marker Expression

The average percentage of immunopositive cells was calculated in four high-power fields per each case. The expression score and the intensity score were determined according to Table 1. The final staining Index was calculated for each case by multiplying its expression score with its intensity score [18].

**Table 1:** Scoring of TGF-β and α-SMA immune-expression; calculation of staining index

Expression score	
0	No positive cells
1	1 to 25% positive cells
2	26 to 50% positive cells
3	51 to 100% positive cells
Intensity score	
0	When there was no staining
1	Where positivity was observed at magnification of 400× only
2	Where the staining was obvious at 100×, but not at 40×
3	In fields where immunopositive cells were seen even at 40×

Staining index: The net result of multiplying the expression and intensity scores

Zero = 0; Low = 1, 2; Moderate = 3, 4; and High = 6 to 9

### Statistical Methods

Statistical analysis of the results was performed using SPSS software. The Shapiro–Wilk test of normality was used to test the normality hypothesis of all continuous variables. Analysis of variance (ANOVA) test was used for the evaluation of the statistical significance of the difference in continuous data for each parameter among the studied groups, followed by the Tukey–Kramer post hoc test for the statistically significant results. Upon comparison between two groups, an unpaired *T*-test was used for the evaluation of the statistical significance of each parameter. For ordinal independent variables, the Kruskal–Wallis test was used for the evaluation of the statistical significance of each parameter among the studied groups, followed by the Tukey–Kramer post hoc test for the statistically significant results. *P*-values ≤ 0.05 were considered statistically significant.

## Results

### Clinical Data

Clinical data for the studied cases was collected as shown in Table 2.

**Table 2:** Clinical data of the studied cases

		PDSCC	MDSCC	WDSCC	OPSCC
Age	Range	50–71	20–71	32–70	47–64
Sex	M	28.60%	62.50%	66.70%	71.40%
	F	71.40%	37.50%	33.30%	28.60%
Site	Lateral border of the tongue	42.90%	25.00%	33.30%	14.30%
	Buccal mucosa	28.50%	25.00%	22.20%	28.60%
	Alveolar mucosa	14.30%	25.00%	33.3%	57.1%
	Floor of the mouth	14.30%	12.50%	11.1%	−
	Palate	−	12.50%	−	−
LN involvement	+	28.60%	25.00%	33.30%	28.60%
	−	28.60%	50.00%	44.40%	42.80%
	N/A	42.80%	25%	22.20%	28.60%

### Histopathological and Immunohistochemical Findings

**Fig. 1:**
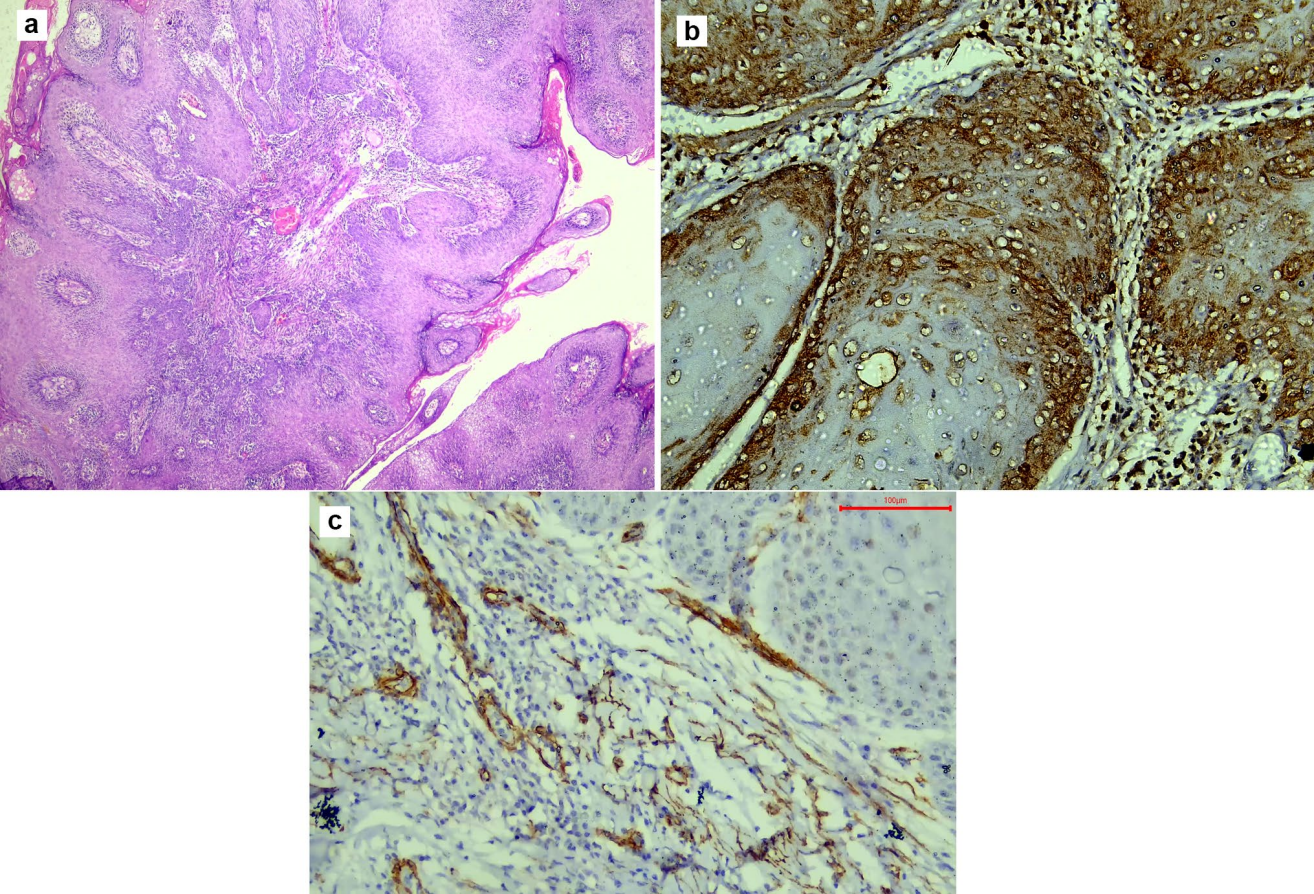
Microscopic images of OPSCC **a** showing a lesion with exo-endophytic growth and interpapillary clefts filled with keratin. Small tumour nests were invading the underlying connective tissue (H&E stain, magnification ×40). **b** Showing strong cytoplasmic expression of TGF-β in the basal cells (immunohistochemistry, magnification ×200). **c** Showing weak focal expression of α-SMA in the stroma under the rete ridges only (immunohistochemistry, magnification ×200)

**Fig. 2:**
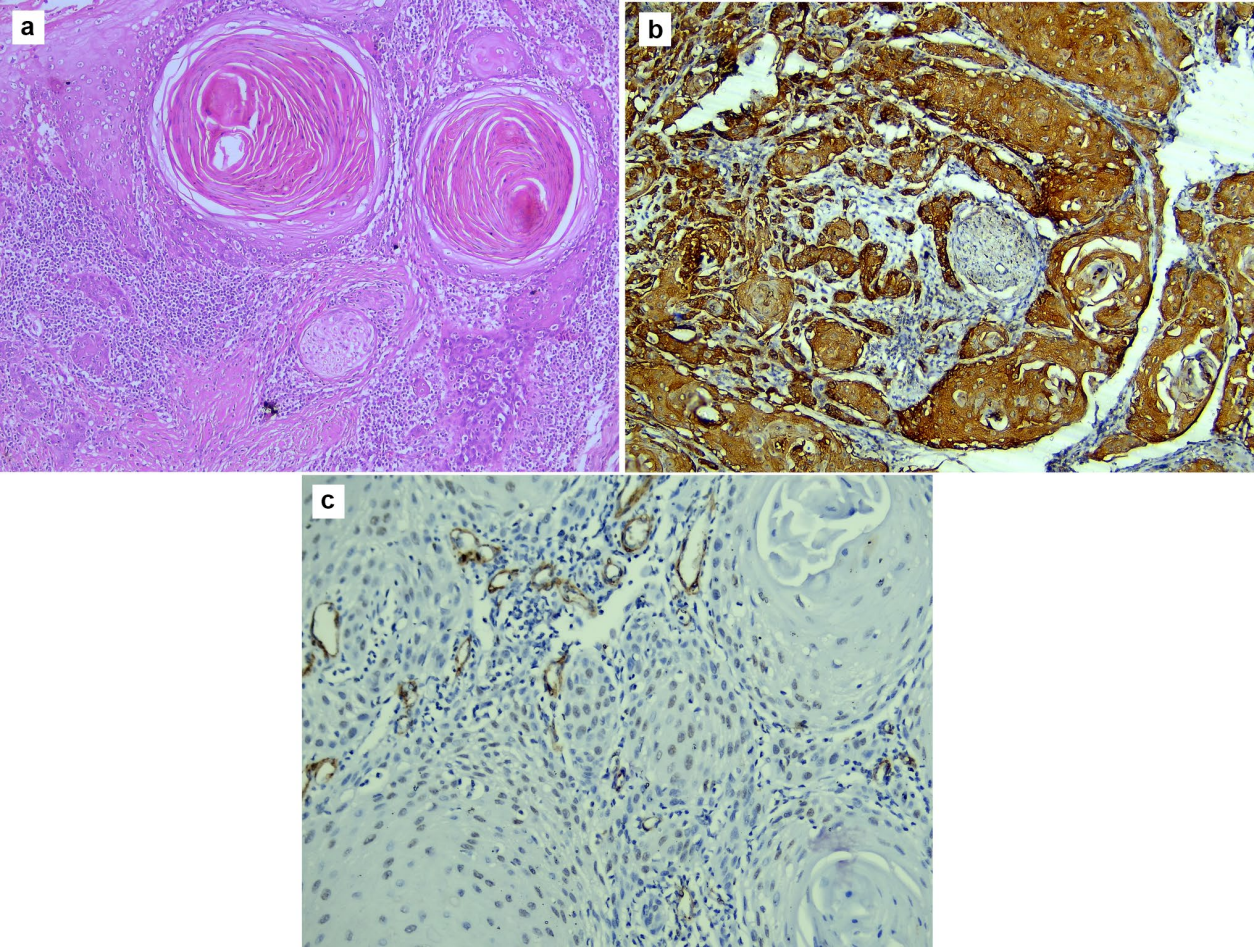
Microscopic images of WDSCC: **a** showing perineural invasion, tumour budding, and increased inflammatory cell infiltrate (H&E stain, magnification ×100). **b** Showing strong diffuse expression of TGF-β; notice the perineural invasion of dysplastic epithelium (immunohistochemistry, magnification ×200). **c** Showing weak focal expression of α-SMA expression in the stroma (immunohistochemistry, magnification ×200)

**Fig. 3:**
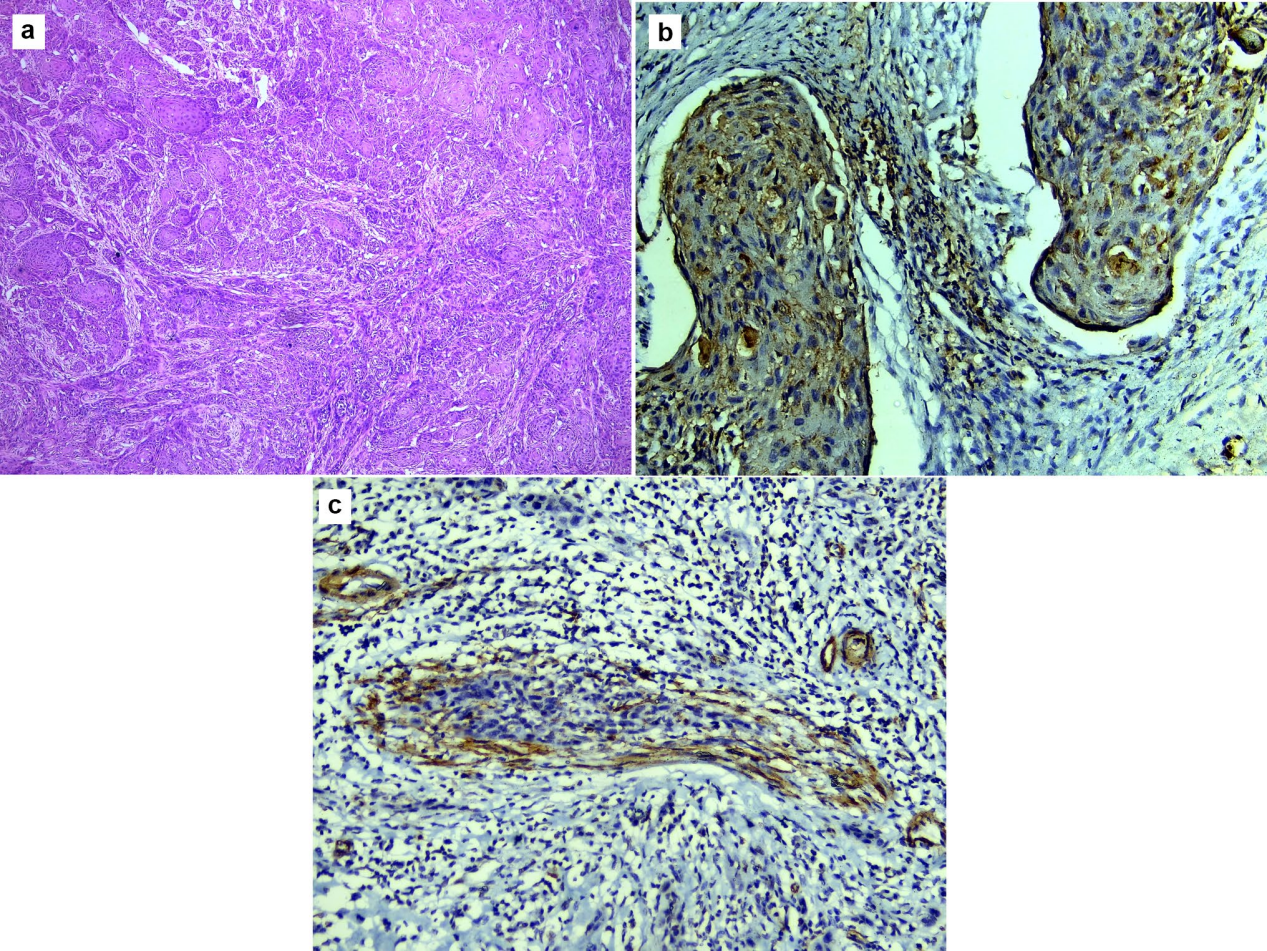
Microscopic images of MDSCC: **a** showing numerous small cell nests (tumour budding), with minimal keratinization invading the connective tissue (H&E stain, magnification ×40). **b** Showing focal TGF-β expression (immunohistochemistry, magnification ×200). **c** Showing α-SMA expression at the invasive front (immunohistochemistry, magnification ×200)

**Fig. 4:**
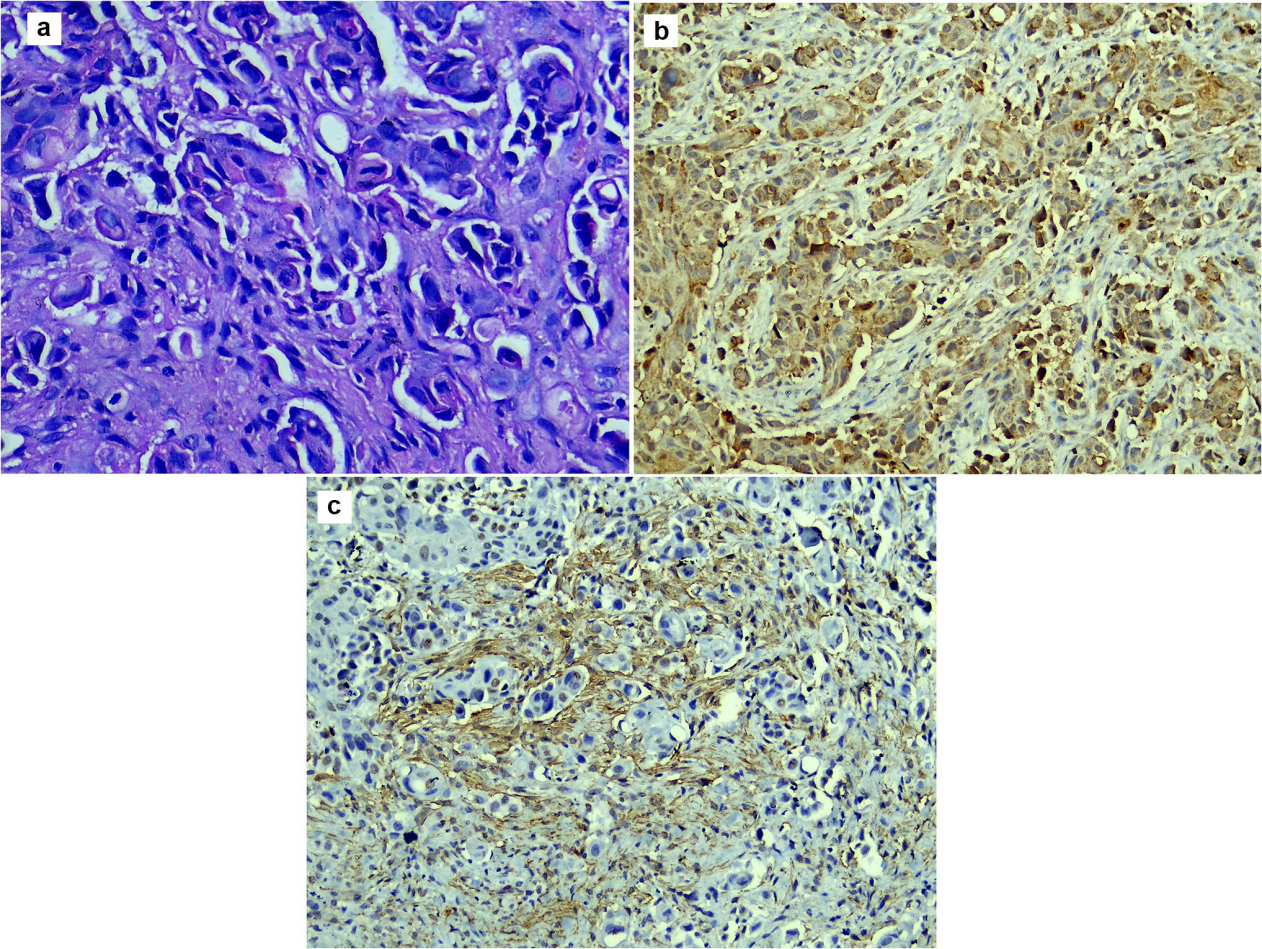
Microscopic picture of PDSCC: **a** showing bizarre-looking nuclei and abnormal mitosis (H&E stain, magnification ×200). **b** Showing focal cytoplasmic expression of TGF-β in epithelium with few nuclear expressions (H&E stain, magnification ×200). **c** Showing strong stromal expression of α-SMA (immunohistochemistry, magnification ×200)

**Fig. 5:**
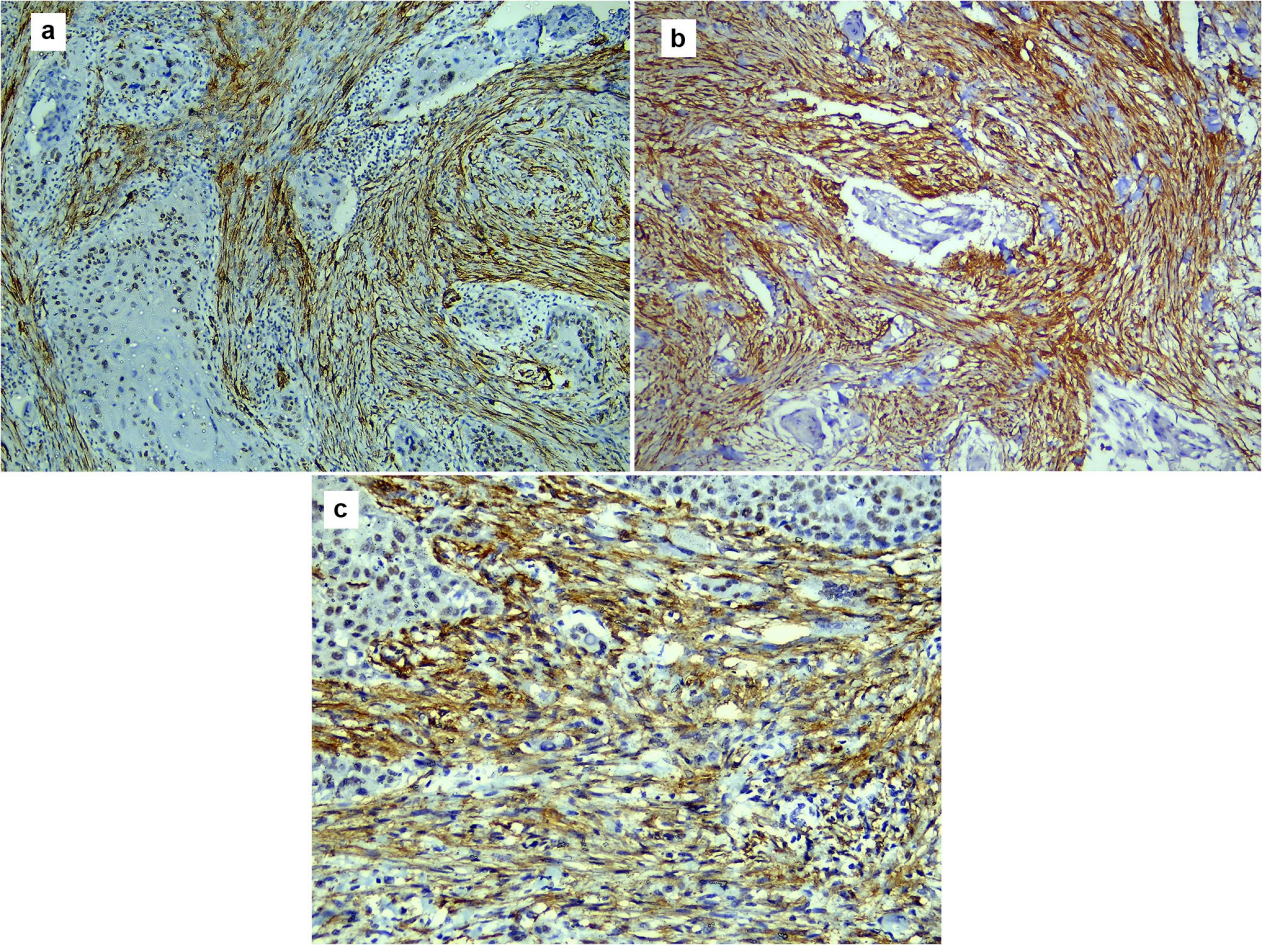
Microscopic pictures of strong diffuse α-SMA expression in the stroma of cases that occurred in the floor of the mouth, with a low TSR: **a** WDSCC case. **b** MDSCC case. **c** PDSCC case (immunohistochemistry, magnification ×200)

Microscopic examination of OPSCC sections stained with H&E revealed a papillary lesion with fingerlike projections. The proliferating epithelium showed frank signs of dysplasia, with an evident invasion into the underlying connective tissue. TGF-β in OPSCC showed strong cytoplasmic expression in the basal cell layer, while α-SMA showed weak focal expression in the stroma just under the rete ridges, as shown in Fig. 1. In WDSCC, the dysplastic epithelial cells could be seen in large nests with numerous keratin pearls. TGF-β was strong diffuse, while α-SMA showed weak scattered expression, as shown in Fig. 2. In MDSCC, the lesions showed a lack of cellular adhesion, less keratin, and nests of smaller size. TGF-β was focal and weak, however, α-SMA expression was demonstrated at the invasive front, as shown in Fig. 3. The dysplastic epithelial cells in PDSCC were invading the underlying connective tissue individually. Signs of dysplasia were very prominent in all cases of PDSCC, including pleomorphism, hyperchromatism, numerous abnormal mitotic figures, and increased mitosis. PDSCC showed focal cytoplasmic expression of TGF-β in the epithelium with some nuclear expression, while it showed strong stromal expression of α-SMA, as shown in Fig. 4. Cases of the floor of the mouth; of the different grades of OSCC, showed the highest stromal expression of α-SMA, as shown in Fig. 5. The histopathological prognostic factors of the collected cases are displayed in Table 3.

**Table 3:** Histopathological prognostic factors of the collected cases

	Tumor budding		Tumor stroma ratio		Perineural/perivascular invasion	
	Evident	Not evident (%)	Low tumor stroma ratio	High tumor stroma ratio (%)	Evident	Not evident (%)
OPSCC	−	100	−	100	−	100
WDSCC	77.80%	22.20	44.40%	55.60	11.10%	88.90
MDSCC	75.00%	25.00	37.50%	62.50	50.00%	50.00
PDSCC	100.00%	0.00	57.10%	42.90	71.40%	28.60

### Statistical Analysis

The comparison of the scoring index of TGF-β in tumour cells among the studied groups was statistically significant. However, the comparison of the scoring index of α-SMA in the stroma was statistically insignificant, as shown in Table 4.

**Table 4:** Mean & median of scoring index of all parameters in all groups and significance of the difference using Kruskal-wall is test with post hoc

		OPSCC	WDSCC	MDSCC	PDSCC
TGF-β in tumor cells	Median	6	9	3	6
	Mean	5.6^a,b,c,d^	8.14^a,b,c^	3.6^b,c,d^	5.3^a,b,c,d^
	SD	1.13	1.46	1.9	1.25
	P-value	0*			
α-SMA in stroma	Median	2	3	3	3
	Mean	2.3	3.14	3	3.14
	SD	0.756	1.46	1.4	1.34
	P-value	0.419^NS^			

*Significant at *p* < 0.05 *NS* not significant

Post hoc test: means sharing the same superscript letter are not significantly different

**Fig. 6:**
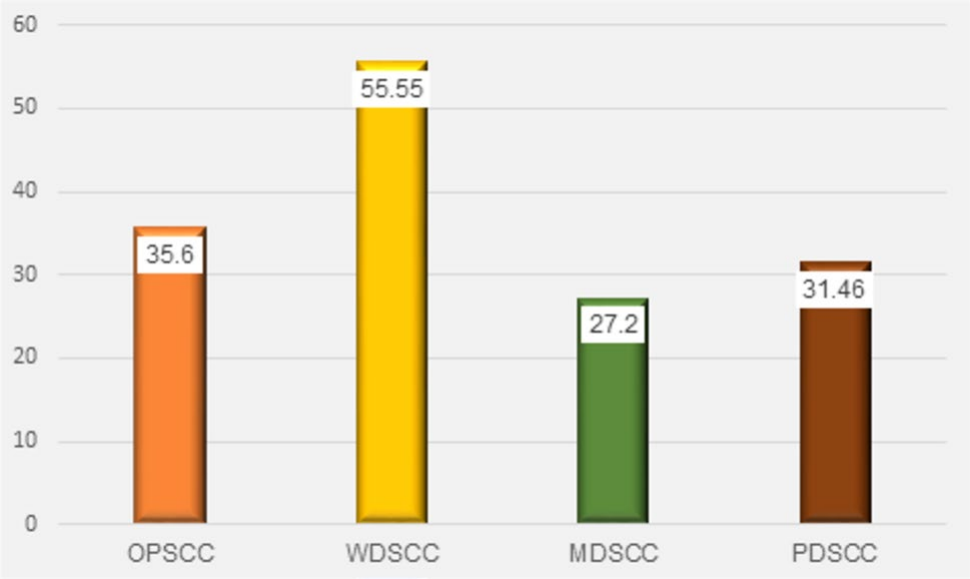
Column chart showing the mean of TGF-β area percent in the tumour cells of all groups

**Fig. 7:**
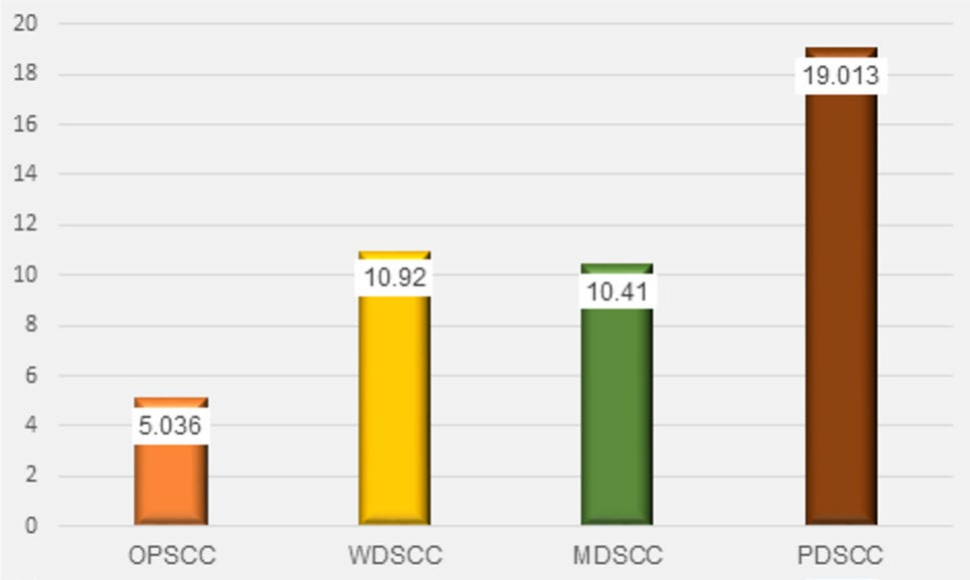
Column chart showing mean of α-SMA area percent in stroma in all groups

The comparison of the area percent of TGF-β in tumour cells among the studied groups was statistically significant, as shown in Fig. 6. As well as, the comparison of the area percent of α-SMA in the stroma was statistically significant, as shown in Fig. 7.

The correlation between TGF-β area percent in tumour cells and α-SMA in stroma in the studied groups is shown in Table 5. The effect of histological prognostic factors on the expression of TGF-β in tumour cells and α-SMA in the stroma is shown in Table 6.

**Table 5:** Pearson correlation coefficient between area percent of TGF-β in tumor cells and α-SMA in stroma in all groups

	R value	Comment
OPSCC	− 0.3521	Although technically a negative correlation, the relationship between expression of TGF-β in epithelium and α-SMA in stroma is only weak*
WDSCC	0.6114	This is a moderate positive correlation, which means there is a tendency for high TGF-β in epithelium expression goes with high α-SMA in stroma (and vice versa)
MDSCC	− 0.0972	Although technically a negative correlation, the relationship between expression of TGF-β in epithelium and α-SMA in stroma is only weak*
PDSCC	0.373	Although technically a positive correlation, the relationship between expression of TGF-β in epithelium and α-SMA in stroma is weak*

**Table 6:** The comparison of mean area percent of each marker in relation to the presence and absence of histological prognostic factors using unpaired *T*-test

	TGF-β in tumor cells (%)	*P*-value*	α-SMA in stroma (%)	*P*-value*
Tumor budding	37.04 ± 15.9	0.45	13.94 ± 9.9	0.025
No tumor budding	37.7 ± 9.67		7.33 ± 4.7	
Low tumor stroma ratio	40.3 ± 16.4	0.23	15.34 ± 11.3	0.023
High tumor stroma ratio	36.1 ± 12.3		8.7 ± 5.6	
Lymph node metastasis	40.37 ± 13.8	0.27	11.96 ± 5.6	0.4
No lymph node metastasis	36.64 ± 13.5		13.14 ± 11.3	

## Discussion

OSCC has various prognoses according to histological grade, subtype, and clinical prognostic factors [19]. OPSCC is an uncommon subtype of OSCC that was postulated to have a favourable prognosis in comparison to conventional OSCC [5]. TME in OSCC has a significant role in tumor initiation, progression, and metastasis due to the presence of critical components such as CAFs and myofibroblasts [8]. Therefore, recently, CAFs staining by immunohistochemistry has been highly recommended to correlate their density with different histological grades and subtypes of OSCC [20]. Accordingly, for a better understanding of the TME in OPSCC and its prognosis, the current study used immunohistochemical staining with TGF-β and α-SMA to compare their expression in OPSCC with that in conventional OSCC. As well as to correlate their expression with histopathological and clinical prognostic factors.

The current study revealed that OPSCC was more common in middle-aged male patients (from 47 to 64 years old). The most common affected sites were the alveolar mucosa, followed by the buccal mucosa, and finally the lateral border of the tongue. The floor of the mouth and the palate were not involved. Lymph nodes were involved in only a small percentage of cases; however, not all cases were examined. These clinical findings were in agreement with [21], who found that OPSCC was more common in males. However, they contradicted some of our findings, where they found that OPSCC was common in older patients with a peak age of 63 years old, and they also found that the palate was the most common site of involvement. Furthermore, [22] contradicted our findings, as they found that OPSCC was more common in females and in older patients ranging in age from 53 to 83 years old. They also found that OPSCC was common in the buccal mucosa, followed by the gingiva, the lower lip, the palate, the tongue, and finally the floor of the mouth.

On the other hand, the different histological grades of conventional OSCC in this study occurred in middle-aged and elderly patients. Males were more commonly affected, except for PDSCC, which was more common in females. This was in contrast to [23] who found that high-grade tumours affected older males, while low-grade tumours were more common in younger females. The most affected site of conventional OSCC was the lateral border of the tongue, followed by the alveolar mucosa and buccal mucosa. The palate was the least likely site to be involved. These clinical findings were in agreement with [7], who reported that the lateral border of the tongue was the most commonly affected site of OSCC. They also found that OSCC of the lateral border of the tongue had the highest mortality rate, which may be in agreement with the findings of the current study, which revealed that most of the cases of the lateral border of the tongue were PDSCC. In the present study, LN involvement was higher in WDSCC in contrast to [24], who found that LN involvement increased with increasing the histological grade. This could be attributed to the missed clinical data in some of our studied cases.

Tumour budding is defined as the presence of single tumour cells or clusters of tumour cells less than 5 cells at the invasive front. Tumour budding at the invasive front indicates that tumour cells have been dissociated from the main tumour mass [25]. A tumour with high tumour budding (more than 10 tumour buds), for example, has a WPOI of 4 or above, which is associated with PDSCC [26]. The TSR plays an important role in the prognosis of OSCC, which is defined as the percentage of tumour tissue in relation to the surrounding stroma. A low TSR is correlated with a worse survival rate, poor lymphocytic response, tumour aggressiveness, advanced stage, and treatment resistance [27].

All OPSCC cases in the current study showed a papillary lesion with POIs 1 and 2. No tumour budding, perineural invasion, or perivascular invasion were seen. Furthermore, high TSR was seen in all cases of OPSCC; this could be attributed to the postulated good prognosis of OPSCC [28] found that there is a correlation among tumour budding and the WOI of the tumour, as tumours with high tumour budding (5–10 tumour buds) have a WOI of 4 or 5 and are often associated with nodal metastasis, whereas tumours with low tumour budding have a WOI of 1 to 3 and are frequently not associated with nodal metastasis. They also found that WPOI and tumour budding have better prognostic values than the histological grade. However, in the current study, WDSCC showed WOI 4 in some cases; in contrast to [28], this can be explained by the detection of tumour budding in more than half of our cases of WDSCC. On the other hand, all cases of PDSCC in this study showed WOI 4, which is in agreement with [28]. As well, PDSCC showed the highest percentage of tumour budding among the three histological grades of OSCC, which correlated with [29], who found that high tumour budding was found in high-grade tumours. They also found that tumour budding was correlated with tumour aggressiveness and metastasis. This could be explained as follows: tumour cells at the invasive front behave more aggressively than cells in the central or superficial part, where they EMT, leading to metastasis and a poor prognosis. Therefore, there is a significant correlation between tumour budding and the prognosis of OSCC [29].

Moreover, PDSCC showed the lowest percentage of a high TSR, which was in the same context as [30], who found that a high TSR was correlated with larger cell nest size and hence related to low-grade tumours. About perineural invasion, PDSCC showed the highest percentage, which correlated with [31], who found that perineural and lymphovascular invasion were correlated with advanced histological grade. In addition [32], found that 70% of cases with perineural invasion were PDSCC, which means that the higher the grade, the more perineural or perivascular invasion will be found.

In the following study, the immunohistochemical staining of TGF-β in OPSCC showed cytoplasmic expression in only the basal and parabasal cells of the dysplastic epithelium, with a high percentage of a high and moderate scoring index. These findings match the findings of [33] who found strong basal and parabasal TGF-β expression in the erythematous form of oral lichen planus, which is one of the oral potentially malignant lesions. This may indicate that the pathological behaviour of OPSCC matches that of the oral potentially malignant lesions. They also found that the expression of TGF-β was similar to that of microinvasive carcinoma, which indicates an excellent prognosis.

On the other hand, the immunohistochemical staining of TGF-β in epithelial cells of WDSCC showed significantly the highest expression in the present study, with a high scoring index in all cases. While MDSCC and PDSCC showed focal expression of TGF-β in the epithelium, the weakest scoring index was in the MDSCC group. These findings were in contrast to [34], who found that high-grade tumours and more aggressive clinical behaviour were associated with increased TGF-β. As well [35], contradicted our study as they reported that the expression of TGF-β, as the main inducer of EMT, increases with the advanced stage of cancer, so the percentage of TGF-β expression should be higher in PDSCC. The low percentage of TGF-β expression in MDSCC and PDSCC in the presented study could be attributed to the discohesiveness of the malignant epithelial cells in these groups, leading to a lower area percent of expression than WDSCC in the fixed frame area.

However, the percentage of TGF-β expression in the stroma was highest in PDSCC, followed by WDSCC, OPSCC, and MDSCC; the difference between the groups was insignificant [36] found that the percentage of TGF-β expression in the stroma increased with poor prognosis for cancer., and this was explained by the secretion of TGF-β by the TME, which acts in a paracrine manner and stimulates protumorigenic microenvironmental changes such as the conversion of activated fibroblasts into CAFs, which in turn leads to a higher histological grade, a poorer prognosis and secretion of TGF-β from other cell types, so it is expected to be found in the stroma.

About α-SMA expression in the stroma, it showed weak scattered cytoplasmic expression in the stroma of OPSCC that was only observed beneath the rete ridges. No OPSCC case showed a high scoring index of α-SMA expression in the stroma. These findings were a match for [37] findings in microinvasive carcinoma. As well as [38] findings, which showed that oral potentially malignant lesions had few myofibroblasts and a weak percentage of α-SMA expression in the stroma. This weak and focal α-SMA expression in the stroma indicates a smaller amount of CAFs and activated myofibroblasts, which may indicate a good prognosis for OPSCC.

On the other hand, the expression of α-SMA in the stroma of WDSCC and MDSCC was focal and moderate, while it showed the highest area percent in the stroma of PDSCC, which was significantly different from its expression in OPSCC only. However, the difference in the mean scoring index of α-SMA in the stroma was insignificant among the four groups. These findings were in correlation with [39] who found that there is no significant difference in α-SMA expression comparing different grades of OSCC. On the contrary [40], found that the highest scoring index of α-SMA expression in the stroma was in PDSCC. In this study, α-SMA expression was seen strongly around the invading malignant cells. This was in accordance with [41], who found that α-SMA expression was found strongly around invading nests, indicating that CAFs were aggregated mostly in these areas, which highlights that there is a strong interaction between CAFs and the invading dysplastic cells.

Interestingly, α-SMA staining in the stroma showed the highest expression in the floor of the mouth cases, regardless of the histological grade. This could be explained by [42], who found that OSCC in the floor of the mouth had a worse prognosis, a higher tendency of local invasion, and cervical lymph node metastasis, leading to a high mortality rate compared with OSCC in other sites in the oral cavity, which leads to an incomplete response to treatment and a lower survival rate.

In our study, α-SMA showed nuclear expression in the dysplastic epithelial cells, with cytoplasmic expression in some of the PDSCC cases. This could be explained by the process of EMT, which leads to the formation of myofibroblasts and CAFs from the dysplastic epithelial cells [43]. The nuclear expression of α-SMA was explained by [44], who used correlative confocal fluorescence and transmission electron microscopy to investigate the nuclear expression of α-SMA. They found that α-SMA aggregates mainly in close proximity to the outer nuclear membrane with many deep nuclear invaginations containing α-SMA-rich cytoplasm, giving the false nuclear expression in immunostaining. They thought that the presence of these invaginations related to the state of cellular differentiation, as they may play a role in signal transduction. Furthermore, they proposed that the changes that happen in the shape of the cell and nucleus cause mechanical forces and the direct transmission of forces through the cytoplasmic and nuclear cytoskeletons. In the current study, PDSCC significantly showed the highest score index and area percent of α-SMA in the epithelium. Upon correlating TGF-β expression in epithelium with α-SMA expression in stroma, we found a moderate positive correlation in the WDSCC group only, which means there is a tendency for high TGF-β in epithelium expression to go with high α-SMA in stroma. These findings were in agreement with [45], who found that increased TGF-β expression correlates positively with increased α-SMA expression in the stroma. This could be explained by the role of TGF-β in EMT and the activation of resident fibroblasts into CAFs and myofibroblasts. Finding this moderate positive correlation in the WDSCC group could only be attributed to the high expression of TGF-β in the epithelium of WDSCC. Our results revealed a significant difference in the mean area percent of α-SMA in stroma between cases with tumour budding and those without tumour budding, with cases with tumour budding having a higher mean area percent of α-SMA in stroma. These findings were in agreement with [46], who correlated the presence of tumour budding with the advanced stage of OSCC. The higher the histological grade of OSCC, the more CAFs and myofibroblasts, leading to a high area percent of α-SMA in the stroma.

Moreover, there was a significant difference in the mean area percent of α-SMA in stroma between cases with a low TSR and those with a high TSR, with cases with a low TSR having a higher mean area percent of α-SMA in stroma. This could be explained by [47], who found that a low TSR was associated with high stromal content and widely dispersed tumour cells, hence more CAFs and myofibroblasts leading to a high percentage of α-SMA in the stroma.

From the previously mentioned points, we concluded that OPSCC has more limited invasion and hence favourable prognosis than conventional OSCC. Moreover, expression of α-SMA in the dysplastic epithelium or its surrounding stroma may indicate bad prognosis of OSCC. As well as, OSCC occurring in the floor of the mouth is proposed to have bad prognosis regardless of its histological grade. Regardless of the histopathological grade, the expression of α-SMA in TME is correlated to the presence or absence of tumor budding and low TSR.

Last, we recommend further molecular investigation of OPSCC using a larger sample size, as well as the other variants of OSCC with correlation with the follow-up period. Modify a histopathological grading system for OSCC to include the histopathological negative prognostic factors.

## Data Availability

The data used to support the findings of the study are included within the article.
